# Constitutive modeling of an electro-magneto-rheological fluid

**DOI:** 10.1038/s41598-022-08549-7

**Published:** 2022-03-17

**Authors:** Deepak Kumar, Somnath Sarangi

**Affiliations:** 1 Department of Mechanical Engineering, Maulana Azad National Institute of Technology Bhopal, Bhopal, Madhya Pradesh 462003 India; 2 Department of Mechanical Engineering, Indian Institute of Technology Patna, Patna, 801103 India

**Keywords:** Mechanical engineering, Aerospace engineering, Engineering

## Abstract

The present article deals with a continuum mechanics-based method to model an electro-magneto-rheological (EMR) fluid deformation subjected to an electromagnetic field. The proposed method follows the fundamental laws of physics, including the principles of thermodynamics. We start with the general balance laws for mass, linear momentum, angular momentum, energy, and the second law of thermodynamics in the form of Clausius–Duhem inequality with Maxwell’s equations. Then, we formulated a generalized constitutive model for EMR fluids following the representation theorem. Later, we validate the model with the results of an EMR rheometer and ER fluid valve system-based configurations. At last, the possible simulation-based velocity profiles are also discussed for parallel plate configuration. As a result, we succeed in providing more physics-based analytical findings than the existing studies in the literature.

## Introduction

In the current scenario, electro-magneto-rheology established a new research direction, which potentially provides fruitful ideas for modern engineering and medical field applications. In line with that, new hybrid electro-magneto-rheological (EMR) fluid systems that couple electro-magneto-mechanical properties of smart fluids^1–3^ are under development in laboratories worldwide. At the same time, there is a specific need to understand the deformation of such smart fluids due to their usage finding in various applications like clutches and brakes in cars, vibration-dampers, and absorbers, lubricating fluids in bearings, smart transducers for medical purposes, etc. In engineering applications, EMR fluids are generally used in hydraulic systems, which are inherently nonlinear. Therefore, their accurate model prediction became so essential that it will directly affect the system performance. The current work is motivated by our previous works on electro-magneto-active (EMA) solids^4–7^. In brief, EMR fluids are colloidal suspensions of polar particles. Similarly, the particles are added as impurities in EMA solids. In EMR fluid, the particles undergo Brownian motion without external fields. While in EMA solids, the filler particles are randomly oriented in the absence of external fields. On the application of external field, the EMR fluid behaves as near-solid but not like a solid. This exceptional nature demarcates the EMR fluid from EMA solid. Thus, thermodynamic pressure is inevitable in the case of EMR fluid, irrespective of whether an external field is applied or not. One may readily appreciate that the constitutive relation of EMR fluid will be governed by the stretch rate, which is time-dependent. Conversely, the EMA solids are considered incompressible hyperelastic, which essentially exhibits the time-independent nature and constitutive relation associated with a Lagrange multiplier to address the incompressibility^8,9^. In the current work, we focus on the constitutive modeling of EMR fluids specifically used in smart hydraulic systems. In general, EMR fluids are characterized by their ability to vary their mechanical properties with an electromagnetic field significantly. EMR fluids are smart, synthetic fluids that change their viscosity from liquid to semi-solid state within milliseconds if a sufficiently strong electric or magnetic field is applied^10,11^. These fluids usually consist of micron-sized electro-magneto-active filler particles dissolved in a non-conducting liquid like mineral or silicone oil. EMR fluids also offer an innovative potential for quick and adaptively controllable electro-magneto-mechanical interfaces in control units when used in suitable devices^12,13^. A coupled electro-magneto-rheological theory is needed to model such EMR fluids within the framework of the continuum mechanics-based approach. Also, the coupled theory must describe the electromagnetic field interaction with the moving deformable media^4,14–16^ such as EMR fluids.

To mention some earlier works on electro-rheological (ER) and magneto-rheological (MR) fluids, Winslow^17^ made an initial attempt to understand the behavior of ER fluids under external gradient. He^17^ investigated an electrically induced fibration of small particles in fluid liquid suspension. Nevertheless, their applicability in fluid system modeling has not been fully explored for many years. Later, the first standardized approach to model such rheological behaviours under external gradients were presented by various researchers, namely, Atkin and Bullogh^18^, Abu-Jdayil and Brunn^19–21^, Rajagopal^10^ along-with Yalamanchili^22^ and Wineman^11^. Herein, they^10,11,22^ adopted the classical continuum mechanics-based approach to model the rheological behaviours of ER fluids under external gradient. Further, some researchers, namely, Conard et al.^23^, and Jordan et al.^24^ also proposed different one-dimensional models for ER fluids. Furthermore, Rajagopal and Wineman^10^ developed three-dimensional models satisfying the appropriate invariance requirements. However, in these models^10^, the electric field was treated as a constant field. In addition to the deformation modeling of ER fluid under external gradient, similar studies on the deformation modeling of MR fluid were also focused by the researchers^12,25,26^ followed by similar approaches. To mention some earlier works on EMR fluids, the theoretical foundations for the analysis of electro-magnetomechanical interactions in EMR fluids were developed in part during the 1990s and 2020s, and a detailed summary may be found, for example, in Hutter et al.^27^ and references therein. In addition, Fujita et al.^1^ investigated a combined electromagnetic field control effect on the rheological properties of a fluid. However, they^1^ have not fully utilized the magnetic field for fine control of the rheological properties. Next, Minagawal et al.^2^ developed a new technique to measure the rheological properties of fluids under electric and magnetic fields. Further, Koyama et al.^3^ compared an EMR effect observed in fluids with the parallel and cross-field systems. Moreover, they^3^ suggested that the parallel-field effect was more significant than the crossed one. At last, we observe that many works on the modeling of ER and MR fluids have been devoted. However, the deformation mechanics dedicated to an EMR fluid continua under an electromagnetic field is still not been reported to the best of our knowledge.

The present article aims to generalize the deformation concept of fluid continua to electro-magneto-rheology in contrast to the existing studies on ER and MR fluids. A constitutive model is developed here for an incompressible isotropic non-Newtonian EMR fluid subjected to an electromagnetic field. To develop the same, a thermodynamically consistent classical continuum mechanics-based approach^14,28^ is adopted. Additionally, the developed model is also experimentally validated in contrast to the existing works^10,11,22^ aimed at the pure theoretical models for smart fluids.

The further part of the paper is organized as follows. In “Fundamentals of electro-magneto-rheology”, a brief review on electro-magnetorheology related to an isotropic EMR fluid continua is summarized. In “Constitutive modeling”, a generalized constitutive relation is developed for an incompressible isotropic non-Newtonian EMR fluid under an electromagnetic field. Next, in “Application to few standard fluid flow situations”, the developed constitutive relation is applied to an ER fluid valve system to validate the same. Further, in “Results and discussion”, different velocity profiles are predicted from the developed constitutive equation for different forms of shear viscosity in the parallel plate configuration. At last, “Concluding remarks” explains about some concluding remarks.

## Fundamentals of electro-magneto-rheology

In this section, a brief review on electro-magneto-rheology related to the deformation of EMR fluid continua is presented, followed by the fundamental laws of physics. These fundamental laws provide initial steps to model the exact electro-magneto-rheological behavior of EMR fluids under an electromagnetic field application.

### Kinematics

Consider an EMR fluid occupying the material space $\beta _0$ in a stress-free configuration. The material point in reference configuration $\beta _0$ is represented by the position vector **X** with respect to an origin. With an application of electromagnetic field, the material deforms and a point **X** in the material occupies a new position $\mathbf{x }=$ $\kappa ({\mathbf{X }},t)$ in the current configuration $\beta$. Wherein $\kappa$ represents an one-to-one deformation mapping. Therefore, the corresponding velocity **v** and acceleration **a** of that material point in three-dimensional Euclidean space, at the instant of time *t* are defined as

1 $$\begin{aligned} {\mathbf{v }=\frac{\partial \mathbf{x } ({\mathbf{X }},t)}{\partial {t}}}, \quad {\mathbf{a }=\frac{\partial ^2 \mathbf{x } ({\mathbf{X }},t)}{\partial {t}^2}}. \end{aligned}$$

The velocity gradient tensor $\mathbf{L }(\mathbf{x },t)$ is represented as

2 $$\begin{aligned} \mathbf{L }=\nabla \mathbf{v }, \end{aligned}$$

wherein $\nabla \mathbf{v }$ represents the gradient operator on velocity field. This velocity gradient tensor $\mathbf{L }(\mathbf{x },t)$ may also be represented in the sum of symmetric part **d** and anti-symmetric part **W** as

3 $$\begin{aligned} \mathbf{L }=\mathbf{d }+\mathbf{W }=\dfrac{1}{2} \left( \mathbf{L }+\mathbf{L }^T\right) +\dfrac{1}{2} \left( \mathbf{L }-\mathbf{L }^T\right) . \end{aligned}$$

### Electromagnetic field equations

At reference configuration $\beta _0$, the fluid continua is assumed as a free of current and electric charge with the time-independent properties. Additionally, we consider the electric field vector **E**, the electric displacement vector **D** and the polarization density **P** as the electric field variables. Similarly, the magnetic field vector **H**, the magnetic displacement vector **B** and the magnetization density vector **M** are considered as the magnetic field variables in the current configuration $\beta$. In condensed matter, these field variables are related as

4 $$\begin{aligned} \begin{aligned} \mathbf{D }=\varepsilon _0 \mathbf{E }+\mathbf{P },\quad \mathbf{B }= \mu _0[\mathbf{H }+\mathbf{M }], \end{aligned} \end{aligned}$$

wherein $\varepsilon _0$ and $\mu _0$ represent the electric permittivity and magnetic permeability of free space, respectively. The electric field variables and the magnetic field variables both satisfy the given Maxwell’s equations^29^ under our constant electric field as well as constant magnetic field application assumption as

5 $$\begin{aligned} \begin{aligned} \nabla \times \mathbf{E }=0, \quad \nabla . \mathbf{D }=0,\quad \nabla \times \mathbf{H }=0, \quad \nabla . \mathbf{B }=0, \end{aligned} \end{aligned}$$

wherein $\nabla \times \mathbf{E }$ and $\nabla . \mathbf{D }$ denote the curl and divergence operators on **E** and **D**, respectively.

### Balance laws

The mechanical balance laws for fluids are expressed in the local form as

#### Conservation of mass

6 $$\begin{aligned} \dfrac{\partial \rho }{\partial t}+\mathrm {\nabla .\left( \rho \mathbf{v }\right) }=0. \end{aligned}$$

#### Conservation of linear momentum

7 $$\begin{aligned} \nabla . \mathbf{S } -\rho \dfrac{\partial \mathbf{v }}{\partial t}+\mathbf{f }_{em}=0, \end{aligned}$$

wherein **S** and $\mathbf{f }_{em}$ denotes the Cauchy stress tensor and the electromagnetic body force (per unit volume), respectively. The generalized electromagnetic body force^29^ $\mathbf{f }_{em}$ in terms of electromagnetic field variables is given as

8 $$\begin{aligned} \begin{aligned} \mathbf{f }_{em} = q\mathbf{E }+\mu _0^{-1}\nabla \mathbf{B }.\mathbf{M }+\nabla \mathbf{E }.\mathbf{P } +\dfrac{\partial }{\partial t}\left( \mathbf{P } \times \mathbf{B }\right) +\nabla .\left[ \mathbf{v } \otimes \left( \mathbf{P }\times \mathbf{B }\right) \right] . \end{aligned} \end{aligned}$$

#### Conservation of angular momentum

9 $$\begin{aligned} \varvec{\varepsilon }:\mathbf{S }+[\mu _0^{-1}\mathbf{M }+\mathrm {\mathbf{v }\times \mathbf{P }}]\times \mathbf{B }+\mathrm {\mathbf{P }\times \mathbf{E }}=\mathbf{0 }, \end{aligned}$$

wherein $[\varvec{\varepsilon }:\mathbf{S }]$ represents $\varepsilon _{ijk}\mathbf{S }_{jk}$ and $\varepsilon _{ijk}$ is a permutation tensor. For the detailed discussion on electromagnetic interaction in deformable continua, we refer to the literature^30–32^ and references therein.

#### Conservation of energy (first law of thermodynamics)

10 $$\begin{aligned} \dfrac{d}{dt}\left( \rho U+\dfrac{1}{2} \rho \mathbf{v }.\mathbf{v } \right) =\nabla .\left( \mathbf{S }.\mathbf{v }\right) +w_{em}, \end{aligned}$$

wherein *U* is the internal energy (per unit mass) and $$w_{em} $$ is the electromagnetic power (per unit volume) in the absence of heat. The general expression of the electromagnetic power $$w_{em} $$ is given as^29^

11 $$\begin{aligned} w_{em}=\mathbf{f }_{em}.\mathbf{v }-\mathbf{M }_e.\dot{\mathbf{B }}+\rho \dfrac{d}{dt}\left( \dfrac{1}{\rho } \mathbf{P }\right) .\mathbf{E }_e, \end{aligned}$$

wherein the superposed dot $\dot{()}$ in the corresponding variable represents the material time derivative. From the Lorentz’s theory of electrons, we have $\mathbf{M }_e= {\mu _0}^{-1}\mathbf{M }+\mathbf{v }\times \mathbf{P }$ and $\mathbf{E }_e=\mathbf{E }+\mathbf{v }\times \mathbf{B }$. Herein, the corresponding terms $\mathbf{M }_e$ and $\mathbf{E }_e$ represent the effective magnetization and the effective electric field, respectively. Now, from Eqs. (6) and (7), the above Eq. (10) may be rewritten as

12 $$\begin{aligned} \begin{aligned} \rho {\dot{U}}=\mathbf{S }:\mathbf{L }+\mathbf{M }_e. \dot{{\mathbf{B }}} +\dfrac{\dot{\rho }}{\rho } \left( \mathbf{E }_e.\mathbf{P }+\mathbf{M }_e.{\mathbf{B }}\right) +\dot{\mathbf{P }}.\mathbf{E }_e. \end{aligned} \end{aligned}$$

#### Clausius–Duhem inequality (second law of thermodynamics)

13 $$\begin{aligned} \mathbf{S }:\mathbf{L }+\rho {\dot{\varphi }}\geqslant 0, \end{aligned}$$

wherein ${\dot{\varphi }}$ term represents the material time derivative of Helmholtz free energy function for a given fluid system. The above representation (13) is a systematic way of expressing the second law of thermodynamics used in the classical continuum mechanics in the absence of heat. This inequality is particularly useful in determining whether the constitutive relation of material is thermodynamically consistent or not.

## Constitutive modeling

In this section, the concepts of an electro-magneto-rheological deformation of fluid continua presented in previous “Fundamentals of electro-magneto-rheology” are utilized to derive the constitutive relation for an EMR fluid under an electromagnetic field.

In order to define our EMR fluid system under an electromagnetic field, we first have to define a Helmholtz free energy function $\varphi$ as^27^

14 $$\begin{aligned} \varphi =U-\dfrac{1}{\rho }\left( \mathbf{E }_e.\mathbf{P }+\mathbf{M }_e.{\mathbf{B }}\right) , \end{aligned}$$

through which a thermodynamic pressure $P_{th}$ is defined for the given fluid system. By substituting (14) and (10) into (13), we obtain an expression of the dissipation inequality given as

15 $$\begin{aligned} \begin{aligned} -\rho {\dot{\varphi }}+\mathbf{S }:\mathbf{L }-\mathbf{M }_e.\dot{\mathbf{B }} -\dot{\mathbf{E }_e}.\mathbf{P }\ge 0. \end{aligned} \end{aligned}$$

Following the principle of Euclidean invariance, we may assume that all relativistic effects as well as the effect of Earth’s spin are neglected and there exist a generalized space vector **k** defined as

16 $$\begin{aligned} \mathbf{k }=\hat{\mathbf{k }}(\rho , \mathbf{d },\mathbf{E }_e,\mathbf{B }). \end{aligned}$$

Based on the above space vector (16), we obtain an expression of ${\dot{\varphi }}$ through the total derivative given as

17 $$\begin{aligned} \begin{aligned} {\dot{\varphi }}=\left( \dfrac{\partial \varphi }{\partial \rho }\right) \left( \dfrac{\partial \rho }{\partial t}\right) +\left( \dfrac{\partial \varphi }{\partial \mathbf{d }} \right) : \left( \dfrac{\partial \mathbf{d }}{\partial t}\right) +\left( \dfrac{\partial \varphi }{\partial \mathbf{E }_e}\right) . \left( \dfrac{\partial \mathbf{E }_e}{\partial t} \right) +\left( \dfrac{\partial \varphi }{\partial \mathbf{B }}\right) . \left( \dfrac{\partial \mathbf{B }}{\partial t}\right) . \end{aligned} \end{aligned}$$

In this regard, it is assumed that an objective part only of these time derivatives contributes to the rate of change of the Helmholtz free energy. By replacing the above ${\dot{\varphi }}$ term in Eq. (15), we have

18 $$\begin{aligned} \begin{aligned} \left( \mathbf{S }: \mathbf{L }-\rho \dfrac{\partial \varphi }{\partial \rho } {\dot{\rho }}\right) - \left( \rho \dfrac{\partial \varphi }{\partial \mathbf{d }} \right) : \dot{\mathbf{d }} -\left( \rho \dfrac{\partial \varphi }{\partial \mathbf{E }_e}+\mathbf{P }\right) .\dot{\mathbf{E }_e} -\left( \rho \dfrac{\partial \varphi }{\partial \mathbf{B }}+\mathbf{M }_e\right) .\dot{\mathbf{B }}\ge 0. \end{aligned} \end{aligned}$$

Using Eq. (6), we may rewrite the above dissipation inequality (18) as

19 $$\begin{aligned} \begin{aligned} \left( \mathbf{S }+\rho ^2 \dfrac{\partial \varphi }{\partial \rho } \mathbf{I }\right) : \mathbf{L }-\rho \dfrac{\partial \varphi }{\partial \mathbf{d }}: \dot{\mathbf{d }} -\left( \rho \dfrac{\partial \varphi }{\partial \mathbf{E }_e}+\mathbf{P }\right) .\dot{\mathbf{E }_e} -\left( \rho \dfrac{\partial \varphi }{\partial \mathbf{B }}+\mathbf{M }_e\right) .\dot{\mathbf{B }}\ge 0. \end{aligned} \end{aligned}$$

In general, the above inequality (19) is expected to hold for real materials at all times and at every fixed point in space for a certain class of admissible thermodynamic processes, i.e., processes compatible with the balance laws and the constitutive response functions. By considering the quantities $\dot{\mathbf{d }},\dot{\mathbf{E }_e}$ and $\dot{\mathbf{B }}$ as independent quantities in the above inequality (19), we obtain the different constitutive laws for an EMR fluid system as

20 $$\begin{aligned} \begin{aligned} \dfrac{\partial \varphi }{\partial \mathbf{d }}=\mathbf{0 },\quad \mathbf{P }=-\rho \dfrac{\partial \varphi }{\partial \mathbf{E }_e}, \quad \mathbf{M }_e=-\rho \dfrac{\partial \varphi }{\partial \mathbf{B }}. \end{aligned} \end{aligned}$$

Now, the reduced dissipation inequality may be re-written as

21 $$\begin{aligned} \begin{aligned} \left( \mathbf{S }+\rho ^2 \dfrac{\partial \varphi }{\partial \rho } \mathbf{I }\right) : \mathbf{L }\ge 0. \end{aligned} \end{aligned}$$

The above inequality (21) represents the Cauchy stress **S** as an isotropic in nature. In this regard, we may define a thermodynamic pressure acting on a homogeneous EMR fluid with an application of electromagnetic field as $P_{th}=\rho ^2 \dfrac{\partial \varphi }{\partial \rho }$. For a given EMR fluid that is not capable of dissipating, i.e., there is no entropy generation, a constitutive law for Cauchy stress tensor in terms of thermodynamic pressure is given as $\mathbf{S }=-P_{th} \mathbf{I }$. Further, we would like to point out that some of our assumptions in the present work are rather based on general observations than on careful experimental evidence. Accordingly, we assume that the considered EMR fluid is non-Newtonian and non-conducting in nature. From the invariance requirements, it follows that $\mathbf{S }: \mathbf{L }=\mathbf{S }: \mathbf{d }$. In addition, the Cauchy stress tensor **S** is an isotropic function of its arguments and has various mini-subspaces. This mini-subspaces span can be utilized for the span required to map other tensors that functionally depend on the tensor **S**. Now, from the representation theorem^33^, we consider that the tensors $\mathbf{d }, \mathbf{E }\otimes \mathbf{E }$ and $\mathbf{H }\otimes \mathbf{H }$ belong to the subspace spanned by the mini-subspaces of tensor **S**. We may here now link our previous physical assumption (isotropic Cauchy stress) that is verified from the relation (21) as well. Hence, the corresponding representation theorem^33^ yields

22 $$\begin{aligned} \begin{aligned}{}&\mathbf{S }=\mathbf{S }(\rho , \mathbf{d },\mathbf{E },\mathbf{H })=\alpha _1 \mathbf{I }+\alpha _2 \mathbf{d }+\alpha _3 \mathbf{d }^2 +\alpha _4 \mathbf{E }\otimes \mathbf{E } +\alpha _5(\mathbf{dE} \otimes \mathbf{E }+\mathbf{E }\otimes \mathbf{dE} ) +\alpha _6(\mathbf{d }^2\mathbf{E }\otimes \mathbf{E }+\mathbf{E }\otimes \mathbf{d }^2\mathbf{E }) +\alpha _7\mathbf{H }\otimes \mathbf{H }\\ & \quad +\alpha _8(\mathbf{dH} \otimes \mathbf{H }+\mathbf{H }\otimes \mathbf{dH} ) +\alpha _9(\mathbf{d }^2\mathbf{H }\otimes \mathbf{H }+\mathbf{H }\otimes \mathbf{d }^2\mathbf{H }), \end{aligned} \end{aligned}$$

wherein $\alpha _{1,2,3 \ldots 9}= f(I_1, I_2,I_3, \ldots I_9)$ are functions of the following invariants that may be represented as

23 $$\begin{aligned} \begin{aligned}{}&I_1= \mathrm {tr} \mathbf{d }, \quad I_2= \mathrm {tr} \mathbf{d }^2, \quad I_3= \mathrm {tr} \mathbf{d }^3, \quad I_4= \mathrm {tr} [\mathbf{E }\otimes \mathbf{E }], \quad I_5=\mathrm {tr} [\mathbf{dE} \otimes \mathbf{E }], \quad I_6= \mathrm {tr} [\mathbf{d }^2\mathbf{E }\otimes \mathbf{E }],\quad I_7= \mathrm {tr} [\mathbf{H }\otimes \mathbf{H }], \\&\quad I_8=\mathrm {tr} [\mathbf{dH} \otimes \mathbf{H }], \quad I_9= \mathrm {tr} [\mathbf{d }^2\mathbf{H }\otimes \mathbf{H }]. \end{aligned} \end{aligned}$$

These functions are to be derived from the micro-mechanics model of the EMR fluids. Physically, such functions have significant potential to include the micro-mechanics-based physics of an EMR fluid. The material modeling of its kind for EMR fluids differs from the literature. For brevity, we are not going towards the micro-mechanics-based combined electro-magneto-mechanical field effect on EMR fluid in detail. But, what we anticipate is that these functional forms, whatever is to be given, may record the least material parameters.

Finally, the above relation (22) combined with (23) represents a physics-based thermodynamically consistent generalized constitutive relation for an EMR fluid under an applied electromagnetic field. Our primary objective of the work was to derive the generalized constitutive relation for EMR fluids following the representation theorem. Aiming the same, the derived relations in the current section are the first of its kind to deal with the combined effect of electromagnetic field on rheological fluids where ER and MR fluids are the special case of the same.

## Application to few standard fluid flow situations

In this section, the developed constitutive relation (22) for an EMR fluid is directly applied to few common fluid flow situations (e.g., shear flows) in order to formulate the associated non-zero components of the stress tensor for each of the considered cases.

**Figure 1:**
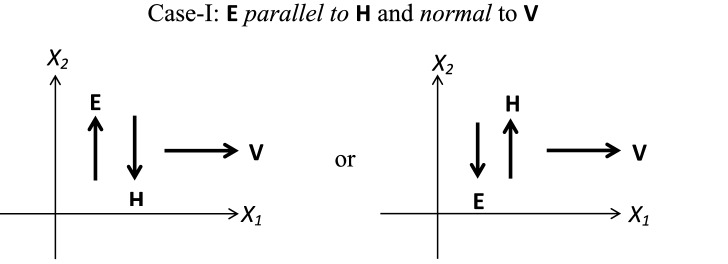
**Case-I:** Applied electric and magnetic fields are mutually parallel and normal to the EMR fluid flow direction.

In line with the shear flow applications, we consider three different standard cases as shown in Figs. 1 (**Case-I**), 2 (**Case-II**) and 3 (**Case-III**), which are discussed in Table 1. In all of the considered fluid flow situations, an EMR fluid flows in the $X_1-X_2$ plane of a Cartesian system. For the given configurations, an EMR fluid is assumed to be isotropic, incompressible, and isothermal. In the first **Case-I**, the applied electric and magnetic fields are mutually parallel and normal to the EMR fluid flow direction. Next, in **Case-II**, the applied electric field is mutually perpendicular to the magnetic field and parallel to the EMR fluid flow direction. At last, in **Case-III**, the applied electric and magnetic fields are mutually perpendicular and normal to the EMR fluid flow direction. In addition, we herein assume that the fluid velocity varies only in one direction significantly, and the other directional variations are negligible.

**Table 1:** Standard fluid flow situations.

Case-I: Applied electric and magnetic fields are mutually parallel and normal to the EMR fluid flow direction	Case-II: Applied electric field is mutually perpendicular to magnetic filed and parallel to the EMR fluid flow direction	Case-III: Applied electric and magnetic fields are mutually perpendicular and normal to the EMR fluid flow direction
For the defined **Case-I** as shown in Fig. 1, the forms of the velocity field vector **v**, electric field vector **E** and the magnetic field vector **H** are obtained as $$\begin{array}{lllllll} \mathbf{v }= \gamma (t) X_2 \mathbf{e }_1, \quad \mathbf{E }= E_0 \mathbf{e }_2, \\ \mathbf{H }= -H_0 \mathbf{e }_2, \end{array}$$ (24) wherein $\gamma (t)$ is the instantaneous shear rate and $E_0$, $H_0$ are the corresponding electric and magnetic field components of an applied electromagnetic field. Now, the corresponding symmetric part of the velocity gradient tensor $\mathbf{d }$ from the definitions (2) and (3) is given as $$\begin{array}{lllllll} \mathbf{d }= \dfrac{\gamma }{2} \mathbf{e }_{12} + \dfrac{\gamma }{2} \mathbf{e }_{21} \end{array}$$. (25)	For the defined **Case-II** as shown in Fig. 2, the forms of the velocity field vector **v**, electric field vector **E** and the magnetic field vector **H** are obtained as $$\begin{array}{lllllll} \mathbf{v }= \gamma (t) X_2 \mathbf{e }_1, \quad \mathbf{E }= -E_0 \mathbf{e }_1, \\ \mathbf{H }= H_0 \mathbf{e }_2, \end{array}$$ (28)	For the defined **Case-III** as shown in Fig. 3, the forms of the velocity field vector **v**, electric field vector **E** and the magnetic field vector **H** are obtained as $$\begin{array}{lllllll} \mathbf{v }= \gamma (t) X_2 \mathbf{e }_1, \quad \mathbf{E }= E_0 \mathbf{e }_2, \\ \mathbf{H }= -H_0 \mathbf{e }_3, \end{array}$$ (31)
Further, the non-zero resulting components of the stress tensor from the generalized constitutive relation (22) are $$\begin{array}{lllllll} S_{11}=\alpha _1 +\alpha _3 \dfrac{\gamma ^2}{4}, \\ S_{22}=\alpha _1+ \alpha _3\dfrac{\gamma ^2}{4}+\alpha _4 {E_0}^2\\ +\alpha _6\dfrac{\gamma ^2E_0}{2}+\alpha _7{H_0}^2 +\alpha _9\dfrac{\gamma ^2H_0}{2}, \\ S_{33}=\alpha _1,\\ S_{12}=\alpha _2\dfrac{\gamma }{2}+\alpha _5 \gamma {E_0}^2+\alpha _8 \gamma {H_0}^2, \\ S_{21}=\alpha _2\dfrac{\gamma }{2}, \\ S_{13}=S_{31}=S_{23}=S_{32}=0. \end{array}$$ (26)	Now, the obtained symmetric part of the velocity gradient tensor $\mathbf{d }$ from (25) may be reused with the above defined field vectors (28) to formulate the non-zero resulting components of the stress tensor from the generalized constitutive relation (22) as $$\begin{array}{lllllll} S_{11}=\alpha _1 +\alpha _3 \dfrac{\gamma ^2}{4}+\alpha _4 {E_0}^2\\ +\alpha _6\dfrac{\gamma ^2 {E_0}^2}{2}, \\ S_{22}=\alpha _1+ \alpha _3\dfrac{\gamma ^2}{4} +\alpha _7 {H_0}^2\\ +\alpha _9\dfrac{\gamma ^2H_0}{2}, \\ S_{33}=\alpha _1,\\ S_{12}=\alpha _2\dfrac{\gamma }{2}+\alpha _8 \gamma {H_0}^2, \\ S_{21}=\alpha _2\dfrac{\gamma }{2}+\alpha _5 \gamma {E_0}^2, \\ S_{13}=S_{31}=S_{23}=S_{32}=0. \end{array}$$ (29)	Next, the obtained symmetric part of the velocity gradient tensor $\mathbf{d }$ from (25) is reused with the above defined field vectors (31) to formulate the non-zero resulting components of the stress tensor from the generalized constitutive relation (22) as $$\begin{array}{lllllll} S_{11}=\alpha _1 +\alpha _3 \dfrac{\gamma ^2}{4}, \\ S_{22}=\alpha _1+ \alpha _3\dfrac{\gamma ^2}{4}+\alpha _4 {E_0}^2\\ +\alpha _6\dfrac{\gamma ^2E_0}{2}, \\ S_{33}=\alpha _1, \\ S_{12}=\alpha _2\dfrac{\gamma }{2}+\alpha _5 \gamma {E_0}^2, \\ S_{21}=\alpha _2\dfrac{\gamma }{2}, \\ S_{13}=S_{31}=S_{23}=S_{32}=0. \end{array}$$ (32)
The corresponding invariants for the defined field vectors (24) that help to express $\alpha _{i}= f(I_1, I_2,I_3, \ldots I_9)$ where $i=1,2,3 \ldots 9$ from their definitions (23) are given as $$\begin{array}{lllllll} I_1=0, \quad I_2=\dfrac{\gamma ^2}{2},\quad I_3=0, \\ I_4={E_0}^2, \quad I_5=0, \quad I_6=\dfrac{\gamma ^2{E_0}^2}{4}, \\ I_7={H_0}^2, \quad I_8=0, \quad I_9=\dfrac{\gamma ^2{H_0}^2}{4}. \end{array}$$ (27)	The corresponding invariants for the defined field vectors (28) that help to express $\alpha _{i}= f(I_1, I_2,I_3, \ldots I_9)$ where $i=1,2,3 \ldots 9$ from their definitions (23) are $$\begin{array}{lllllll} I_1=0, \quad I_2=\dfrac{\gamma ^2}{2},\quad I_3=0, \\ I_4={E_0}^2, \quad I_5=0, \quad I_6=\dfrac{\gamma ^2{E_0}^2}{4}, \\ I_7={H_0}^2, \quad I_8=0, \quad I_9=\dfrac{\gamma ^2{H_0}^2}{4}. \end{array}$$ (30)	The corresponding invariants for the defined field vectors (31) that help to express $\alpha _{i}= f(I_1, I_2,I_3, \ldots I_9)$ where $i=1,2,3 \ldots 9$ from their definitions (23) are $$\begin{array}{lllllll} I_1=0, \quad I_2=\dfrac{\gamma ^2}{2},\quad I_3=0, \\ I_4={E_0}^2, \quad I_5=0, \quad I_6=\dfrac{\gamma ^2{E_0}^2}{4}, \\ I_7={H_0}^2, \quad I_8=0, \quad I_9=0. \end{array}$$ (33)

**Figure 2:**
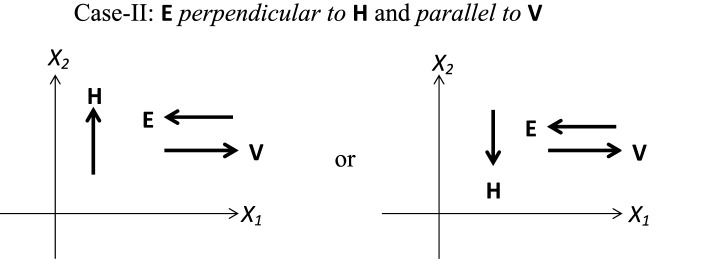
**Case-II:** Applied electric field is mutually perpendicular to magnetic field and parallel to the EMR fluid flow direction.

**Figure 3:**
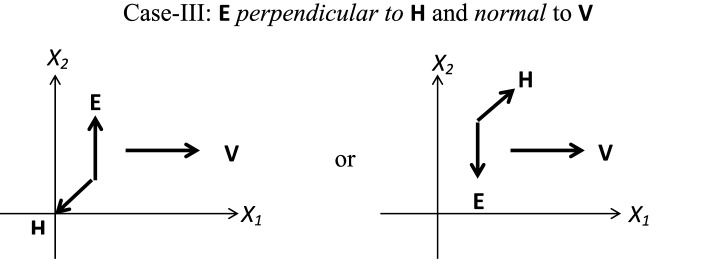
**Case-III:** Applied electric and magnetic fields are mutually perpendicular and normal to the EMR fluid flow direction.

## Results and discussion

In this section, an analytical model for an ER fluid valve system configuration consistent with^34^ is modeled through the developed constitutive relation (22) in order to validate the same. In addition, the different velocity profiles are also predicted from the same constitutive equation (22) for different forms of shear viscosity in the parallel plate configuration.

### Experimental validation of the constitutive relation (ER fluid case)

**Figure 4:**
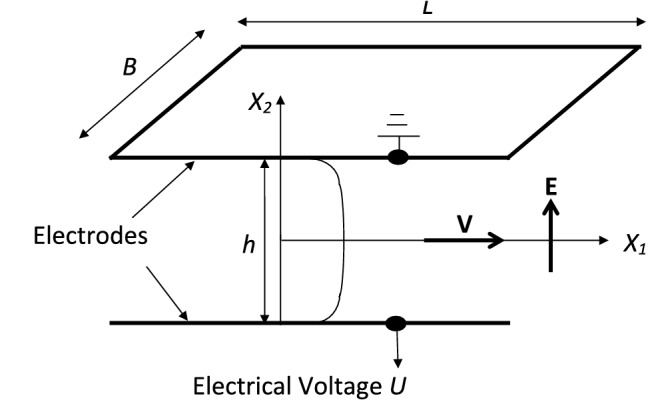
System configuration of a flat channel ER valve.

To check the validity of constitutive relation (22) derived in “Constitutive modeling”, we compare our theoretical result obtained from (22) at zero magnetic field with the existing experimental work performed by Kamelreiter et al.^34^. Kamelreiter et al.^34^ designed an ER valve geometry to measure the field-dependent yield stress of a typical ER fluid. This ER valve consists of a flat (or annular) channel of height *h* formed by two electrodes as shown in Fig. 4. The electrodes have the dimensions as length *L* and width *B*. The ER fluid in the channel is assumed to be isotropic, incompressible, and isothermal. Herein, the volume flow was driven by the pressure difference between inlet and outlet pressures. With an application of electrical voltage *U* at a single electrode and by earthening the other electrode, an electric field was generated that changes the rheological properties of the ER fluid.

We begin our theoretical analysis of the same ER valve geometry used by Kamelreiter et al.^34^ as shown in Fig. 4. To obtain an analytical expression of the field-dependent shear stress model $$S_{12}$$, we first have to choose the appropriate functional forms of $$\alpha _2$$ and $$\alpha _5$$ based on the definitions of invariants (27) through ($$26)_4$$. In line with that, we may choose the corresponding appropriate functional dependency of $$\alpha _2$$ and $$\alpha _5$$ based on the simplest algebraic functions for the considered ER valve configuration as

34 $$\begin{aligned} \begin{aligned} \alpha _2=\alpha _2(I_1,I_2, \ldots I_6)=K_1\gamma ^2 {E_0}^2, \quad \alpha _5=\alpha _5(I_1,I_2, \ldots I_6)=K_2\gamma ^2 {E_0}^2. \end{aligned} \end{aligned}$$

Now, for the above simplest functional forms of $$\alpha _2$$ and $$\alpha _5$$, the simplified component of the Cauchy stress tensor (22) through ($$26)_4$$ at $$H_0=0$$ is represented as

35 $$\begin{aligned} \begin{aligned} S_{12}=K_1\dfrac{\gamma ^3}{2} {E_0}^2 +K_2\gamma ^3 {E_0}^4. \end{aligned} \end{aligned}$$

The above Eq. (35) represents a physics-based thermodynamically consistent analytical expression of the field dependent shear stress model derived from (22) for an ER fluid under an applied electric field. In addition, Kamelreiter et al.^34^ had developed an analytical model similar to our (35) for the same ER valve geometry as shown in Fig. 4 as

36 $$\begin{aligned} \begin{aligned} S_{12}=a_1 E_0+ a_2 {E_0}^2 + a_3 {E_0}^3, \end{aligned} \end{aligned}$$

wherein $a_1$, $a_2$ and $a_3$ are the constant parameters, which were obtained from the measurements for the characteristic behaviorof a typical ER fluid.

**Figure 5:**
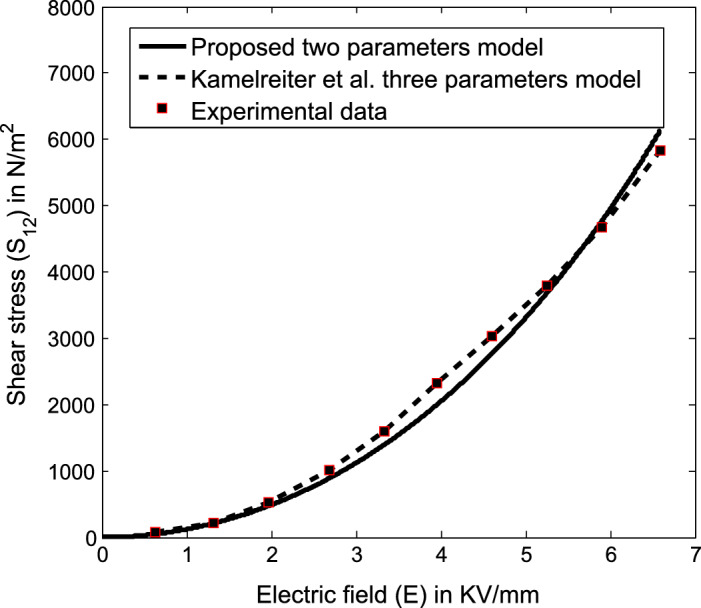
Comparison of the model (35) derived from (22) with the kamelreiter et al. model (36) as well as the experimental data^34^ on the field dependent shear (yield) stress of a typical ER fluid.

To compare the proposed model (35) derived from (22) with the model (36) as well as the experimental data of a typical ER fluid from^34^, we plot the same on Fig. 5 for $K_1=1$ and $K_2=119.68$ at a fixed strain-rate $\gamma =1$ based on the geometrical dimensions of the system configuration.

We here note that the theoretical predictions of the considered ER valve system through the model (35) are consistent with the experimental data^34^. In addition, the success of the proposed model (35) in characterizing the considered system is evident in two aspects. Firstly, it may produce a simple closed-form analytical solution to the problem. Secondly, a comparison of the proposed model (35) with the experimental data^34^ requires the determination of only two material constants: $K_1$ and $K_2$ even for an extensive range of the applied field. Whereas, another model (36) that compares favorably with the experimental data do so by fitting three parameters: $a_1$, $a_2$ and $a_2$ that lack, in most cases, any physical interpretation at all and also demand considerable computation. Also, the proposed model (35) considers zero slope condition at a null electric field which is evident from the experimental data. On the other side, Kamelreiter et al.^34^ model does not ensure the zero slope at null electric field condition. For more clarity, relations (35) and (36) are to be re-looked, especially the derivative with respect to the applied field.

### Experimental validation of the constitutive relation (EMR fluid case)

**Figure 6:**
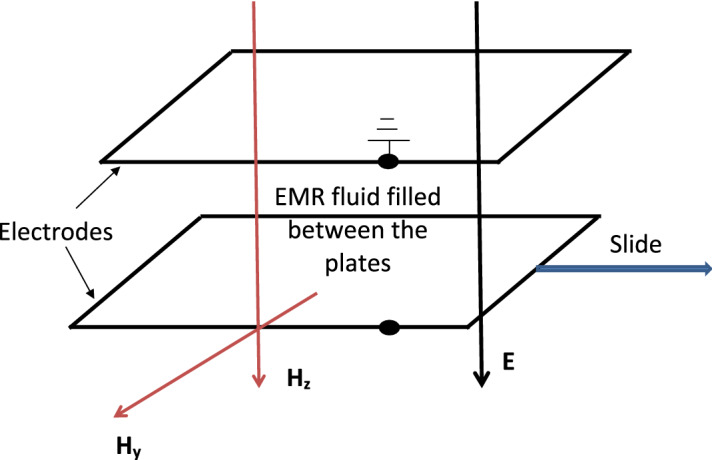
System configuration of an EMR rheometer.

To further access the validity of constitutive relation (22), we compare the analytical findings at a non-zero electromagnetic field case with the experimental work performed by Koyama et al.^3^ They^3^ carried out rheological measurements of EMR fluid by the use of a parallel-plate rheometer equipped with electrodes and magnetic coils as shown in Fig. 6. In this rheometer, the magnetic coils were placed above and below the plates with a distance of 6 mm between the cores of two coils.

The electric field was applied to the system by the electrodes attached to the surfaces of the plates. At the same time, the magnetic field was generated through the motion of the coils toward the plates. The rheological measurements were taken as the stress increase induced by electric and magnetic fields.

#### Parallel-field condition ($\mathbf{E } \parallel \mathbf{H }$)

Firstly, we model the above EMR fluid-based rheometer configuration used by Koyama et al.^3^ as shown in Fig. 6 in parallel-field condition. We choose an appropriate functional forms of $$\alpha _2$$, $$\alpha _5$$ and $$\alpha _8$$ based on the definitions of invariants (27) through ($$26)_4$$ before obtaining the expression of the field-dependent shear stress model $$S_{12}$$. In line with that, we may choose the functional dependency of $$\alpha _8$$ similar to $$\alpha _2$$ and $$\alpha _5$$ in (34) based on the simplest algebraic function as

37 $$\begin{aligned} \begin{aligned} \alpha _8=\alpha _8(I_1,I_2, \ldots I_6)=\dfrac{K_3\gamma ^2 (1+E_0/2)}{H_0}. \end{aligned} \end{aligned}$$

Now, the simplified component of the Cauchy stress tensor (22) through ($$26)_4$$ is represented as

38 $$\begin{aligned} \begin{aligned} {S_{12}}=K_1\dfrac{\gamma ^3}{2} {E_0}^2 +K_2\gamma ^3 {E_0}^4+K_3\gamma ^3 (1+E_0/2){H_0}. \end{aligned} \end{aligned}$$

The above Eq. (38) represents a physics-based electromagnetic field dependent shear stress model derived from (22) for an EMR fluid system under an applied electromagnetic field in parallel-field condition.

**Figure 7:**
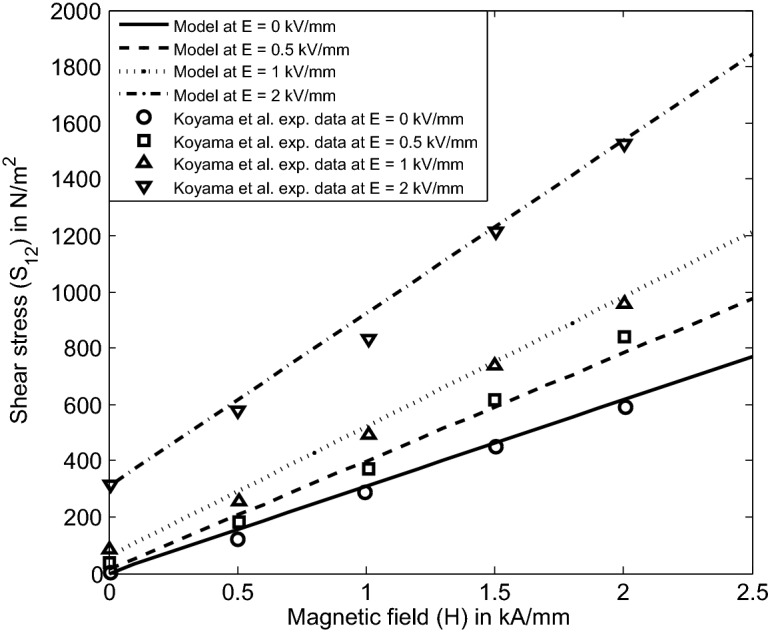
Comparison of the model (38) derived from (22) with Koyama et al.^3^ experimental data on the shear stress of a typical EMR fluid in parallel-field condition.

To compare the model (38) with the experimental data of a typical EMR fluid from^3^ in parallel-field condition, we plot the same on Fig. 7 for a fixed strain-rate $\gamma =2.8$ based on the system configuration shown in Fig. 6. In the plot 7, the shear stress $S_{12}$ is plotted against applied magnetic field intensity *H* at different applied electric field intensities. Herein, an increase in the slope of the lines under higher electric fields indicates the ‘synergistic effect’ of the electric and magnetic fields on the EMR effect. This effect is taken care through our field dependent material constant $\alpha _8$ mentioned in (37) that contains a simplest possible algebraic form. In addition, the theoretical predictions of the model (38) obtained from the constitutive relation (22) are consistent with the experimental data^3^. The model (38) is validated with the experimental data^3^ for the given set of parameters $K_1 = 5$, $K_2 = 0.25$, $K_3 = 14$. The parameters $K_1$, $K_2$ and $K_3$ are obtained from the evident linear nature.

#### Crossed-field condition ($\mathbf{E } \perp \mathbf{H }$)

**Figure 8:**
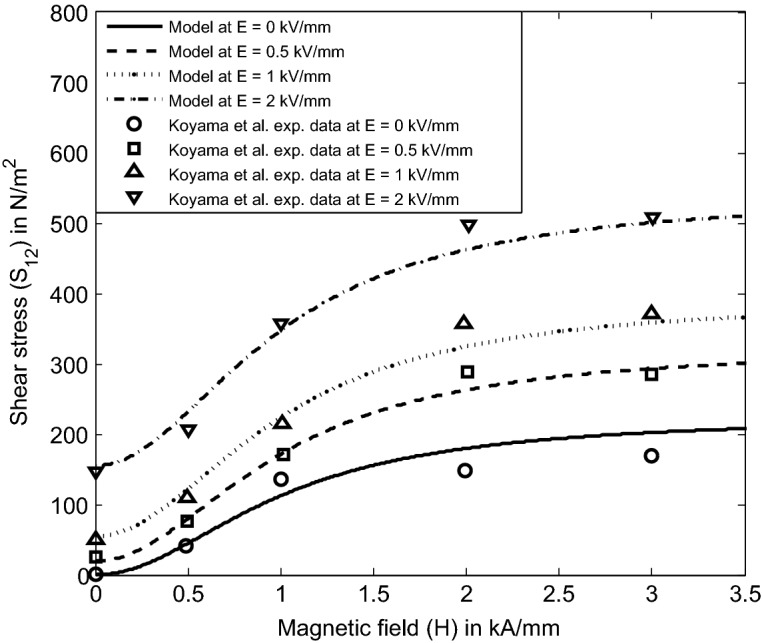
Comparison of the model (40) derived from (22) with Koyama et al.^3^ experimental data on the shear stress of a typical EMR fluid in crossed-field condition.

Secondly, we model the considered EMR fluid-based rheometer configuration used by Koyama et al.^3^ as shown in Fig. 8 in crossed-field condition. We choose the functional forms of $$\alpha _2$$ and $$\alpha _5$$ based on the definitions of invariants (27) through ($$26)_4$$ to obtain the field-dependent shear stress model $${S_{12}}$$ in crossed-field condition as

39 $$\begin{aligned} \begin{aligned} \alpha _2=\alpha _2(I_1,I_2, \ldots I_6)=K_1\gamma ^2 E_0 \sqrt{E_0}, \quad \alpha _5=\alpha _5(I_1,I_2, \ldots I_6)=K_2\gamma ^5 \left[ \dfrac{{H_0}^2 (2+ \sqrt{E_0})}{ {E_0}^2 (1+{H_0}^2) } \right] . \end{aligned} \end{aligned}$$

Now, the simplified component of the Cauchy stress tensor (22) through ($$26)_4$$ is given as

40 $$\begin{aligned} \begin{aligned} {S_{12}}=K_1\dfrac{\gamma ^3}{2} E_0 \sqrt{E_0} +K_2\gamma ^6 \left[ \dfrac{{H_0}^2 (2+ \sqrt{E_0})}{ 1+{H_0}^2 } \right] . \end{aligned} \end{aligned}$$

The above Eq. (40) represents a physics-based electromagnetic field-dependent shear stress model derived from (22) for an EMR fluid system under an applied electromagnetic field in crossed-field condition. The analytical findings of the model (40) are compared now with the experimental data of a typical EMR fluid from Ref.^3^ in the crossed-field condition in Fig. 8. The shear stress $S_{12}$ is plotted here against applied magnetic field strength *H* at different applied electric field intensities for a fixed strain rate $\gamma =2.8$ based on the experiment^3^. In the plots, the stress reaches a nearly constant value after a particular magnetic field strength for each electric field strength. This saturation indicates that the clusters (enhancement of particle aggregation) induced by the cross-field condition in an EMR fluid system significantly affect the resistance against shear deformation compared to the parallel-field condition. In addition, the analytical predictions of the model (40) obtained from the constitutive relation (22) are consistent with the experimental data^3^. At last, one may here note that the consistency between the model (40) and the experimental data^3^ is obtained using the same set of parameters used in parallel-field condition with the same material composition.

### Possible simulational results

**Figure 9:**
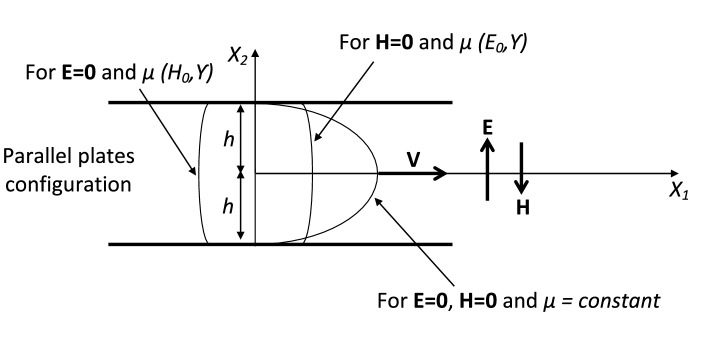
Different velocity profiles for flow between two parallel plates configuration.

In current discussion, consider the effect of the electric and magnetic field components $E_0$ and $H_0$ in each of the considered cases. Herein, the in-plane stress components $S_{11}, S_{22}, S_{12},$ and $S_{21}$ depend on $E_0$ and $H_0$ through the corresponding functions of $\alpha _{i}= f(I_1, I_2,I_3, \ldots I_9)$ where $i=1,2,3 \ldots 9$. Additionally, it may be seen from the relations (26), (29) and (32) with (23) that $\alpha _{1,2,3 \ldots 9}= f(I_1, I_2,I_3, \ldots I_9)$ can be regarded as functions of ${E_0}^2, {H_0}^2$, and $\gamma ^2$. Now for each of the considered cases, we represent the shear rate equation in the generalized shear form as

41 $$\begin{aligned} \begin{aligned} S_{12}= \mu ({E_0}^2, {H_0}^2, \gamma ^2) \gamma (t), \end{aligned} \end{aligned}$$

wherein $\mu ({E_0}^2, {H_0}^2, \gamma ^2)$ represents the shear viscosity function. Herein, $\gamma =\dfrac{dv}{dX_2}$ and some of the $\alpha _i$ terms represent the non-Newtonian nature of EMR fluid. Some of the $\alpha$ terms arise from the interaction of the shear flow and the component of the electric field in the direction normal to the flow direction. From the standard observations^10,11^, we have $S_{12}=-PX_2$. Wherein, *P* is the pressure gradient, and it helps to represent some of the $\alpha _i$ terms, correspondingly. Next, by substituting $S_{12}=-PX_2$ in the above expressions of $S_{12}$ for each of the considered cases, we may easily obtain the differential equations for the velocity $v(X_2)$ in terms of the parameters *P*, $E^2$, $H^2$, and $\gamma ^2$, etc. Now, consider an EMR fluid compassed between two parallel plates having a distance 2*h* apart as shown in Fig. 9.

In the given configuration, a constant electric field $$\mathbf{E }=E_0 \mathbf{e }_2$$ and a constant magnetic field $$\mathbf{H }=-H_0 \mathbf{e }_2$$ is applied perpendicular to the plates. The EMR fluid flow is assumed to be rectilinear in the direction parallel to the plates. For the given fluid flow similar to **Case-I** in “Application to few standard fluid flow situations” situation and the corresponding shear stress expression of $$S_{12}$$ from ($$26)_4$$ with the condition $$S_{12}=-PX_2$$, we may easily obtain the velocity profiles of EMR fluid. Some of the corresponding velocity profiles are predicted successfully for different forms of shear viscosity like $$\mu =constant$$, $$\mu =\mu (E_0, \gamma )$$ and $$\mu =\mu ( H_0, \gamma )$$ as shown in Fig. 9. Additionally, the simulational results shown in Fig. 9 are also consistent with the existing results^10,11^ at zero magnetic field condition.

In summary, we may control the EMR fluid flow situations and play with the corresponding theoretical parameters for the given situation through a specific choice of the relationships between $S_{12}$, $E^2$, $H^2$, and $\gamma ^2$ from the standard experimental evidence. At last, these mathematical arguments will directly help us in the design and development of the different EMR fluid actuators for various engineering and medical field applications.

### An additional comment

From (26), (29) and (32) for each of the considered case, we note that the shear response in **Case-III** is independent of an additional magnetic field application. However, for the remaining **Case-I** and **Case-II**, this additional magnetic field application significantly affects the EMR fluid deformation. Based on this observation, we may comment that an electric or magnetic field normal to fluid flow direction with different plane does not affect the shear flow deformation in EMR fluids.

## Concluding remarks

In the present paper, we develop a thermodynamically consistent generalized constitutive relation (22) for an incompressible isotropic non-Newtonian electro-magneto-rheological (EMR) fluid under an electromagnetic field. The developed constitutive relation (22) relates the Cauchy stress tensor **S** with the stretch rate tensor **d**, electric field vector **E** and magnetic field vector **H**. Next, the relation (22) is applied to study the most common fluid flow situations in modern system configurations and validated with an ER fluid valve system. Later on, the different velocity profiles are also predicted from the same for different forms of shear viscosity in the parallel plate configuration. We strongly believe that applying a magnetic field on an ER flow may convert the steady flow to an unsteady flow and vice versa. Such kind of precise control of the flow field is a subject of separate work. It is a relevant scientific problem with a wide application in biology, soft robotics, and discussed application domains in the introduction section of the manuscript.

The major contribution of the present study is an attempt to generalize the deformation concept of fluid continua to electro-magneto-rheology in contrast to the existing works on ER and MR fluids. Moreover, the previous works are also obtainable from the same as a special case. In addition, an analytical model (35) proposed from (22) is succeed in two ways. Firstly, it produces a simple closed-form solution to the problem. Secondly, it requires a lesser number of material parameters that have a certain physical basis for defining the same phenomenon compared to other existing models (36), that lack, in most cases, like for a large range of the applied field. At last, the proposed study will enrich the physical understanding of the EMR fluids like other systems of interest, which are under development in laboratories worldwide.
